# The landscape of chimeric antigen receptor T cell therapy in breast cancer: Perspectives and outlook

**DOI:** 10.3389/fimmu.2022.887471

**Published:** 2022-07-22

**Authors:** Hao Zhang, Shuangli Zhu, Wanjun Deng, Rui Li, Haiting Zhou, Huihua Xiong

**Affiliations:** Department of Oncology, Tongji Hospital, Tongji Medical College, Huazhong University of Science and Technology, Wuhan, China

**Keywords:** chimeric antigen receptor, CAR-T cell, immunotherapy, breast cancer, tumor microenvironment

## Abstract

Chimeric antigen receptor-T (CAR-T) cell therapy is a revolutionary adoptive cell therapy, which could modify and redirect T cells to specific tumor cells. Since CAR-T cell therapy was first approved for B cell-derived malignancies in 2017, it has yielded unprecedented progress in hematological tumors and has dramatically reshaped the landscape of cancer therapy in recent years. Currently, cumulative evidence has demonstrated that CAR-T cell therapy could be a viable therapeutic strategy for solid cancers. However, owing to the immunosuppressive tumor microenvironment (TME) and heterogenous tumor antigens, the application of CAR-T cell therapy against solid cancers requires circumventing more challenging obstacles. Breast cancer is characterized by a high degree of invasiveness, malignancy, and poor prognosis. The review highlights the underlying targets of CAR-T cell therapy in breast cancer, summarizes the challenges associated with CAR-T cell therapy, and proposes the strategies to overcome these challenges, which provides a novel approach to breast cancer treatment.

## Introduction

According to the most recent global cancer statistics in 2020, breast cancer has overtaken lung cancer to become the most commonly diagnosed cancer, with approximately 2.3 million newly diagnosed cases and 680,000 deaths (1). Breast cancer severely threatens women’s health because of its high malignancy and extremely poor prognosis. Breast cancer is categorized into the following subtypes based on the expression level of estrogen receptor (ER), progesterone receptor (PR), and human epidermal receptor 2 (HER2): basal‐like breast cancer, luminal A, luminal B, triple-negative breast cancer (TNBC), and human epidermal growth factor receptor 2 (HER-2) amplified subtype (2). Surgery, chemotherapy, and radiotherapy remained the mainstream treatments for breast cancer (3). Fatal complications such as damage to normal breast tissues, recurrence, and metastasis after treatment seriously limit the effect of breast cancer treatment (4, 5). The continuous clinical application of targeted therapies such as trastuzumab has led to a better prognosis and fewer adverse reactions in HER2-positive breast cancer patients (6). However, its overall therapeutic effect is still limited due to the molecular specificity of targeted therapies, and alternative treatments are urgently required.

The tumor microenvironment (TME) consists of tumor cells, immune cells, mesenchymal cells, and secreted chemokines and cytokines, which jointly regulate the physiological process of tumor cells (7). In recent decades, the regulatory mechanisms of the TME in tumorigenesis have been elucidated, and tumor-infiltrating immune cells play a significant role in the TME. Immunotherapy based on immune checkpoints such as programmed death-1/programmed death ligand-1 (PD-1/PD-L1) and cytotoxic T-lymphocyte-associated antigen 4 (CTLA4), has been extensively applied in multiple tumors. The suppression of immune checkpoints can block the immunosuppressive signals in immune cells and activate them to recognize and destroy tumor cells (8). Immunotherapy has achieved satisfactory clinical therapeutic effects in lung cancer, melanoma, and renal cell carcinoma, which has revived the field of cancer treatment (9).

**Figure 1:**
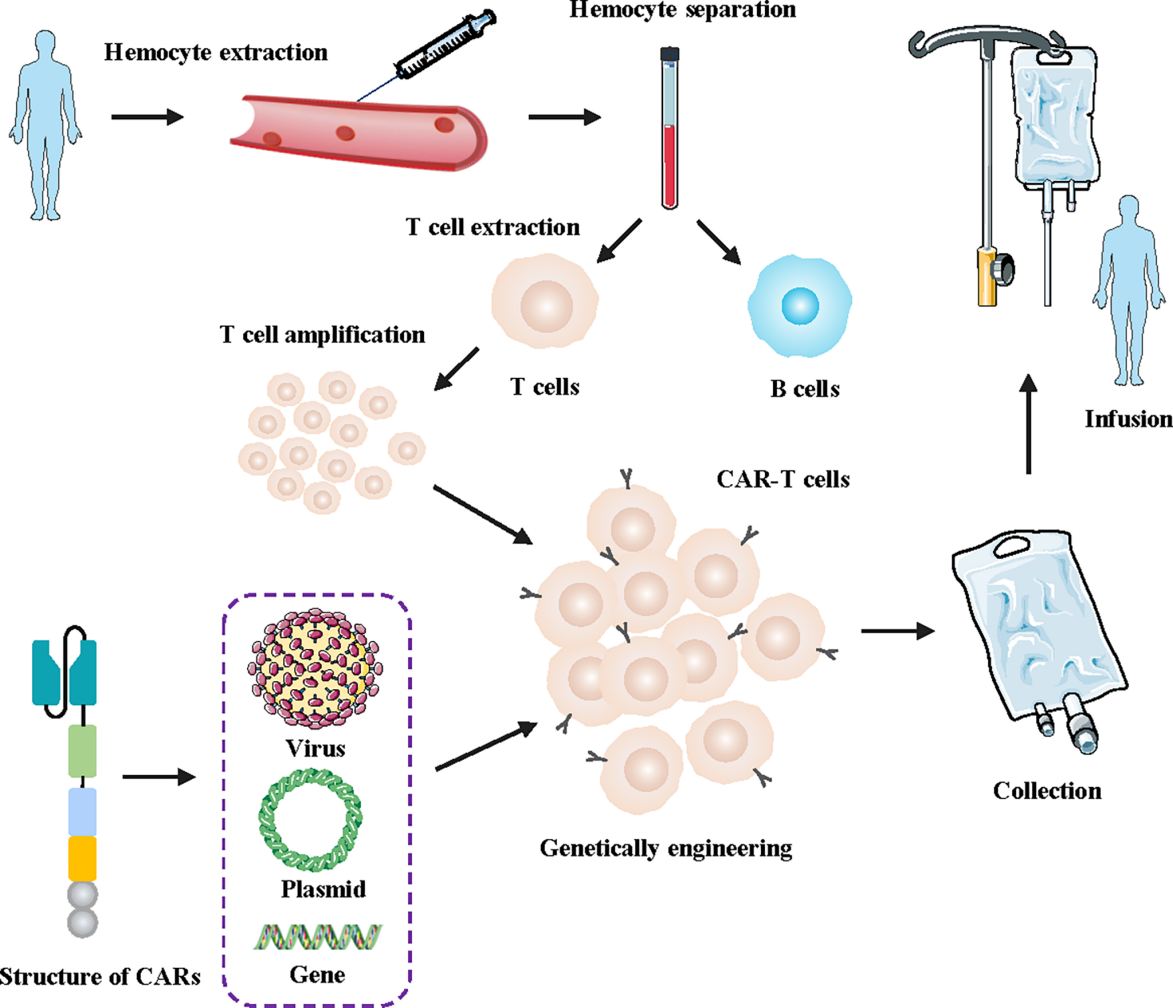
Flowchart for manufacturing engineered CAR-T cells. First, a blood sample was taken from the patient. Secondly, T cells were isolated and collected from the human blood samples. Then, the lentivirus was transfected into the T cells genome of the patients, facilitating the T cells to express artificially modified CARs. Finally, the designed CAR-T cells were massively amplified *in vitro* and subsequently injected into tumor patients.

**Figure 2:**
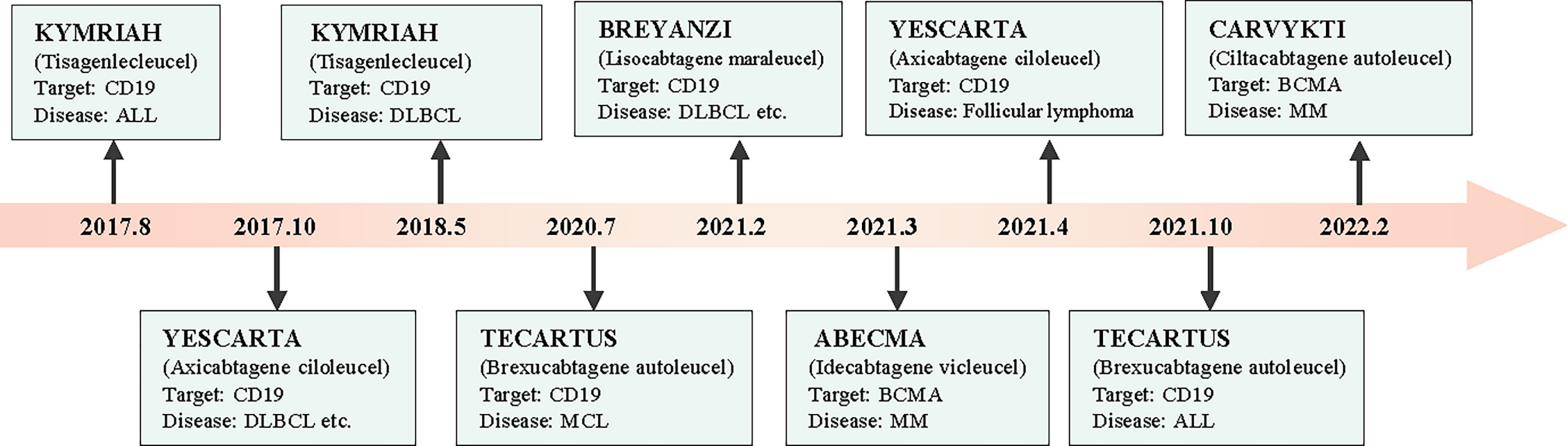
The process of FDA-approved CAR-T cells for tumor treatment.

Chimeric antigen receptor T (CAR-T) cells are artificially engineered T cells that express a synthetic tumor cell-specific receptor on their surface. The preparation process of CAR-T cells is shown in **Figure 1**. T cells were initially isolated from peripheral blood mononuclear cells and transfected with a lentivirus to express CAR. The modified CAR-T cells were subsequently amplified *in vitro* and reinfused back into the patients (10). The CAR-T cells can recognize specific tumor antigens and activate an immune response ultimately eliminating tumor cells (11). CAR-T cell therapy has achieved impressive success against hematologic tumors (12). Since 2017, the FDA has approved six types of CAR-T cells for hematologic tumors (13). (**Figure 2**) The considerable effects of CAR-T cell therapy against hematological malignancies have facilitated its application in solid tumors, including breast cancer. In this review, we introduced the novel CAR-T cell engineering strategy, summarized the potential targets and clinical trials in breast cancer, and discussed the challenges and engineering strategies of CAR-T cell therapy.

## The construction of CARs

**Figure 3:**
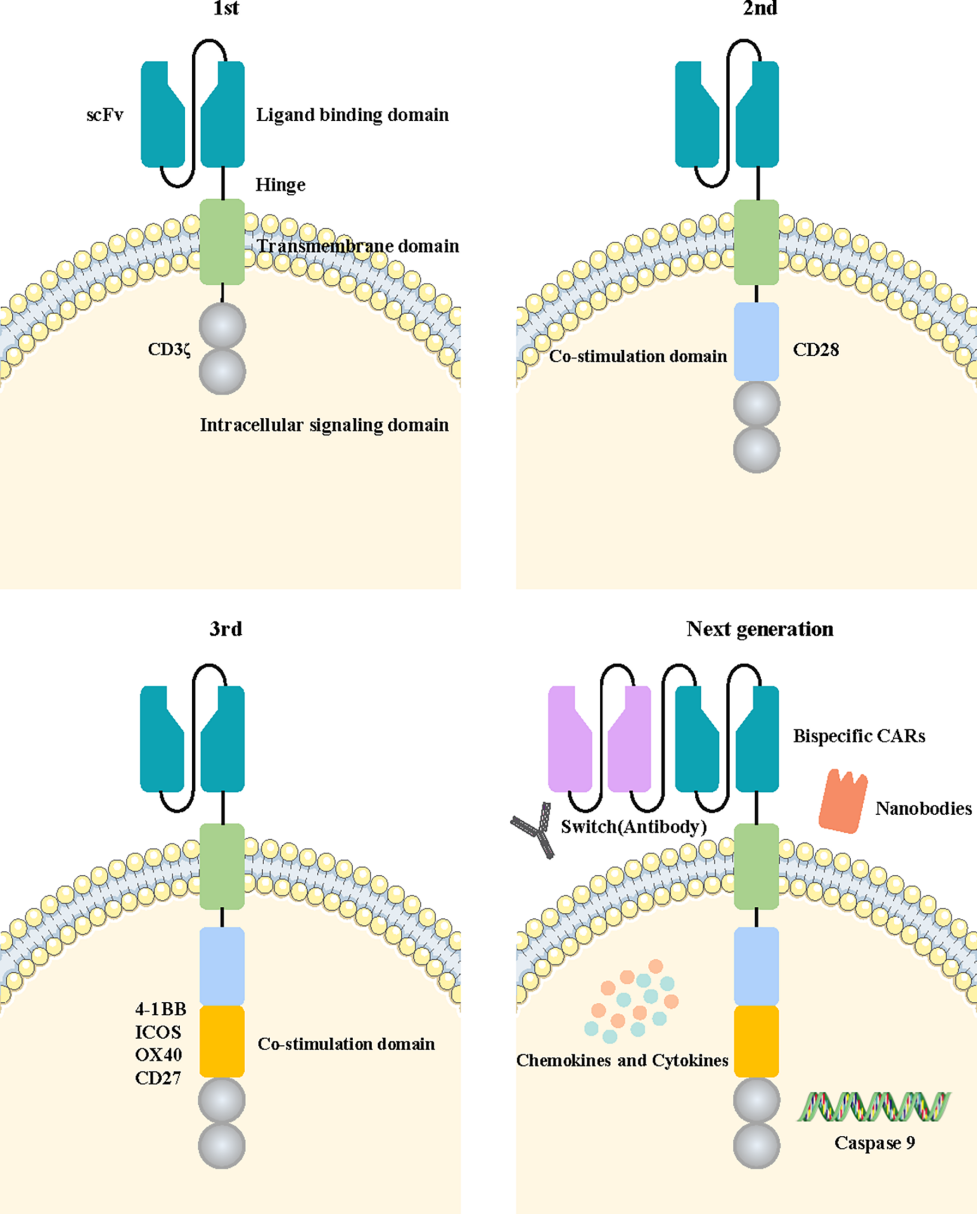
Fundamental structure diagram of a CAR-T cell and the development flowchart from the first-generation to the new-generation CAR-T cell. The fundamental structure of CAR-T cells is composed of extracellular tumor antigen-binding domains, hinge domains, transmembrane domains, and intracellular signaling domains. The first generation of CAR-T cells contained only a CD3ζ intracellular signaling domain. The second or third generation of CAR-T cells added one or more costimulatory molecules based on the previous generation. The next generation of CAR-T cells applied a variety of new engineering strategies, including bispecific CARs, the switch, nanobodies, caspase 9, and the cytokine pathway.

CARs are divided into four main components based on their structure and function, including an antigen-binding domain, a hinge domain, a transmembrane domain, and an intracellular signaling domain (14). The diagrams of the specific structure patterns are shown in **Figure 3**. Optimization of the four domains of CARs during their development can effectively increase their effectiveness and safety in tumor treatment.

The antigen-binding domain is located on the extracellular membrane of CAR-T cells, and mainly plays the role of recognizing the tumor antigen and transducing the recognized antigen signal into the cell (15). The antigen-binding region is a single-chain variable fragment (scFv) composed of a variable heavy chain and light chain of antibodies linked by Gly4Ser peptide, the most common linker in CARs (16). The scFv sequence is usually part of a monoclonal antibody derived from mice or humans. Currently, smaller natural monoclonal antibodies (nanobodies) are also being designed using the scFv sequences (17). This domain recognizes cell tumor-specific antigens and activates T cells, which are independent of major histocompatibility complex (MHC) molecules. It provides a method to overcome immune escape due to the downregulation of MHC molecules in tumor cells (18). The recognition ability of the antigen-binding region and affinity of tumor cells in CAR-T cells directly affect the antitumor effect.

The hinge region is responsible for connecting the extracellular antigen binding domain and the transmembrane region of the cell membrane. It provides more flexibility for the antigen-binding domain to cope with spatial barriers in binding to tumor cells so that CAR-T cells can identify and interact with tumor cells more easily (19).

The transmembrane domain connects with the intracellular and extracellular domains of CAR-T cells and fixes the basic construction of CARs into the cell membrane. Type I proteins are the main component of transmembrane regions such as CD3ζ, CD4, CD28, or CD8α (20, 21). Savoldo et al. found that the CARs transmembrane region containing CD28 has a more stable structure than that containing CD3ζ (22).

The intracellular signal domain is the intracellular localization component of CARs, which usually consists of an activation domain and costimulatory domains. The identification signals of tumor antigens were transmitted to CAR-T cells, activating the intracellular signal domain, and prompting T cells to destroy tumor cells. Most of the activation domains of CARs are derived from CD3ζ immunoreceptors based on tyrosine activation motifs (22). However, the activation signals conveyed by CD3ζ alone are not sufficient to induce a durable immune response in T cells (23). Therefore, costimulatory regions such as OX40, CD27, CD28, 4-1BB, or ICOS are introduced into the structure of CARs. The activation of the costimulatory region was found to dramatically promote the antitumor effect and persistence of CAR-T cells by generating cytokines such as IL-2 (24). The CD27 molecule was confirmed to enhance the killing effect of Trop2-targeted CAR-T cells and to prolong their survival time in breast cancer (25).

## The development of CAR-T cell therapy

With advances in biotechnology, the construction of CAR-T cells has evolved over several generations. The general development process is illustrated in **Figure 3**.

The first-generation CAR-T cells transmit activation signals only through the intracellular region of the CD3ζ domain. They have shown limited efficacy in clinical trials due to a lack of costimulatory signaling, leading to more rapid CAR-T cell death (26). The costimulatory signal domain 4-1BB or CD28 was added into the second generation of CAR-T cells based on the construction of the previous generation. Combined activation of the two signals significantly improved the tumor-killing efficacy and persistence of CAR-T cells. Multiple clinical trials have confirmed that second-generation CAR-T cells targeting CD19 achieved significant clinical efficacy in B-cell acute lymphoblastic leukemia (B-ALL) treatment (27, 28). Moreover, the third generation of CAR-T cells with an additional costimulatory signaling molecule was designed to further enhance the activation ability of CAR-T cells. Costimulatory signaling molecules such as CD27, CD28, ICOS, 4-1BB, and OX40 were commonly used in this generation of CAR-T cells, providing superior antitumor efficacy to their predecessors (29). The third generation of CAR-T cells has become the main widely used technology in the construction of CAR structures. NKG2D CAR-T cells with CD27 or 4-1BB costimulatory signaling molecules could promote the expansion and self-enrichment of CAR-T cells without the presence of IL-2, effectively enhancing the ability to recognize and eliminate breast cancer (30). EGFR‐targeted CAR‐T cells containing CD28, 4‐1BB and CD3ζ costimulatory signaling molecules showed a strong inhibitory effect on tumors (31).

In addition, some novel strategies have started to be carried out to identify CAR-T cell therapies with a better therapeutic effect and minimum adverse reactions. It was found that CD28 can promote cytokine secretion, 4-1BB can increase T cell proliferation, and CD27 can enhance T cell survival. The fourth generation of FRα-targeted CAR-T cells containing the costimulatory domains CD28, 4-1BB, and CD27 demonstrated superior therapeutic efficacy in breast cancer due to the o combined benefits of the three costimulatory domains (32).

Switch-based recombinant dual-function antibody engineering has been developed to address safety concerns associated with CAR-T cell therapy. The structure of the switch includes a Fab molecule that binds specifically to tumor antigens and a peptide epitope that specifically binds to CAR-T cells. CAR-T cells only bind to the peptide epitopes of the switch but not to endogenous tissues or antigens on tumor cells. Hence, the recognition and activation processes are severely dependent on the existence of the switch. This switch strategy could reduce the occurrence of adverse reactions by controlling CAR-T cell activity and cytokine release with the same effect compared with traditional CAR-T treatment (33).

The design of two ligand-binding domains in single-stranded CAR structures is a strategy for the more efficient identification of tumor cells. The CARs can recognize two distinct tumor antigens, either of which is adequate to activate T cells. This activation pattern markedly improves the efficiency of tumor cell recognition (34). In Yang et al.’s study, a bivalent tandem CAR (TanCAR) was designed to target both CD70 and B7-H3, which enhanced antitumor functionality and improved the problem of antigenic heterogeneity and variant in breast cancer (35).

The signal transmission domain, the new structure domain in current CARs, maybe another important signal to activate CAR-T cell function in addition to the costimulatory domain. Kagoya et al. have designed a novel generation of CAR-T cells which add a new signaling molecule domain compared to the traditional CAR-T cells. The signal domain was constructed from the IL-2 receptor β-chain and STAT3 binding tyrosine-X-X-glutamine (YXXQ) motif. These novel CAR-T cells could improve proliferation, antitumor capacity, and persistence compared to traditional CAR-T cells through the activation of JAK kinases and the STAT3/STAT5 transcription factor signaling pathways (36).

## Therapy targets of CAR-T cells in breast cancer

In solid tumors, the construction of CAR-T cells is more complex, and recognition of targeted tumor-specific antigens is an important challenge for CAR-T cell therapy. Over the years, several tumor surface antigens have been determined as promising therapeutic targets for CAR-T cell therapy in breast cancer. In the next sections, we summarize some recent advances of targets in CAR-T cells for breast cancer. Moreover, the summarized targeted information is presented in **Table 1**.

**Table 1:** The summary of targeted of CAR-T cell therapy in breast cancer.

Targets	Targets site	Experimental model	Research Progress	Researcher
HER2	Tumor	*In vitro*/SCID mice model	HER2-targeted CAR-T cells recognized and eliminated trastuzumab-resistant tumor cells.	Gábor et al. (37)
	Tumor	*In vitro*/NSG mice	CAR-T cells penetrate the tumor matrix against HER2 antibody-resistant tumors.	Szöőr et al. (38)
	Tumor	*In vitro*/NSG mice	CAR-T cells is a prospective treatment in breast cancer brain metastases patients.	Saul et al. (39)
HER3/4	Tumor	*In vitro*/Balb/c nude mice	CAR-T cells can damage breast cancer with HER family expression and overcome HER2-targeted therapy resistance.	Zuo et al. (40)
EGFR	Tumor	*In vitro*/SCID mice model	The potential of EGFR CAR-T therapy for TNBC was demonstrated.	Xia et al. (31)
	Tumor	*In vitro*/nude mice	CAR-T is a promising treatment strategy for TNBC patients with high EGFR expression.	Liu et al. (41)
MSLN	Tumor	*In vitro*/NCG mice	CAR-T cells significantly inhibited the proliferation of MLSN - positive breast cancer.	Zhang et al. (42)
ICAM1	Tumor	*In vitro*/NSG mice	CAR-T cells have high therapeutic potential against ICAM1-positive TNBC tumors.	Wei et al. (43)
AXL	Tumor	*In vitro*/NSG mice	CAR-T cell are a promising therapeutic strategy against TNBC.	Wei et al. (44)
MUC1	Tumor	*In vitro*/NSG mice	CAR-T cells have high therapeutic potential against tMUC1-positive TNBC tumors with minimal damage.	Zhou et al. (45)
GD2	Tumor	*In vitro*/NSG mice	CAR-T is a promising novel approach for GD2-positive breast cancer, especially in disseminated and metastasis tumor cells.	Seitz et al. (46)
FRα	Tumor	*In vitro*	The feasibility of FRα-targeted CAR-T cells therapy was confirmed in breast cancer.	Luangwattananun et al. (32)
PD-L1	Tumor	*In vitro*/C57BL/6 mice	The chPD1 T cells can reduce the tumor burden in breast cancer and release cytokines.	Parriott et al. (47)
	Tumor	*In vitro*/NSG mice	CAR-T cells can trigger the expression of PD-L1 on target cells, and enhance the cytotoxicity of PD-L1 CAR-T cells.	Bajor et al. (48)
PTK7	Tumor	*In vitro*/NSG mice	PTK7-targeted CAR-T cells significantly prevented the growth of breast cancer.	Jie et al. (49)
Trop2	Tumor	*In vitro*/NCG mice	CAR-T cells enhanced the CAR-T cell tumor-killing effect.	Chen et al. (25)
SLC3A2	Tumor	*In vitro*/NSG mice	SLC3A2-targeted CAR-T cell is a novel, efficacious, and potentially safe approach for tumor cell therapies.	Pellizzari et al. (50)
B7-H3	Tumor	*In vitro*/NSG mice	A low dose of SAHA significantly enhanced the antitumor activity of B7-H3-targeted CAR-T cells in breast cancer.	Lei et al. (51)
CD70	Tumor	*In vitro*/NSG mice	TanCAR-T cells targeting CD70 and B7-H3 exhibit enhanced antitumor functionality in breast cancer.	Yang et al. (35)
VEGFR 2/3	Tumor/vascular endothelial cells	*In vitro*/nude mice	VEGFR-2/3 CAR-T cells showed cytotoxicity against tumor cells and umbilical vein endothelial cells.	Xing et al. (52)
TEM8	Tumor/vascular endothelial cells	*In vitro*/SCID mice	TEM8-targeted CAR-T cells enhanced the secretion of cytokines and killed tumor cells and endothelial cells.	Byrd et al. (53)
NKG2DLs	Tumor	*In vitro*/NSG mice	Self-enriched CAR-T cells effectively recognized and eliminated TNBC cell lines.	Han et al. (30)
αvβ6	Tumor	*In vitro*/NSG mice	αvβ6-targeted CAR-T cells exhibited strong cytotoxicity to breast cancer cells.	Whilding et al. (54)
CD32A^131R^	Antibody Fc fragment	*In vitro*	CD32A^131R^ CAR-T cells recognize and damage cetuximab-bound tumor cells.	Caratelli et al. (55)
ROR1	Tumor	*In vitro*/NSG mice	Oxaliplatin and anti-PD-L1 synergistically improved ROR1-targeted CAR-T cell anti-tumor ability.	Srivastava et al. (56)

### HER2

Human epidermal growth factor receptor 2 (HER2), one of the most widely studied molecules in breast cancer, is elevated in 20-25% of breast cancer patients. The overexpression of HER2 is related to higher rates of metastasis and recurrence in breast cancer (57). Since the FDA approved trastuzumab in 1998, specific therapies with monoclonal antibodies have revolutionized the mainstream treatment concept for HER2-positive breast cancer. Although HER2-targeted therapies have been widely used in breast cancer patients and achieved good therapeutic results, drug resistance still limits their therapeutic effects in patients (58). The constructed HER2-targeted CAR-T cells can actively recognize tumors and achieved better efficacy and safety in a clinical trial of sarcoma (59, 60). Gábor et al. designed HER2-targeted CAR-T cells with trastuzumab-derived scFv and a CD28 costimulatory domain. The results showed that complete tumor remission was achieved within 57 days in these trastuzumab-resistant breast cancers when only 7% of CAR-T cells consisted of the T cells. The results confirmed that a small quantity of CAR-T cells can have a strong antitumor effect on the anti-HER2 antibodies-resistant xenografts (37). In another study of trastuzumab-resistant breast cancer, HER2-targeted CAR-T cells could infiltrate the core region of the tumor globule, showing tumor cell cytotoxic activity, whereas anti-HER2 antibodies failed. Moreover, CAR-T cells can penetrate the tumor matrix and eradicate tumors in trastuzumab-resistant breast cancer xenografts. This study demonstrated that CAR-T cells can effectively overcome antibody therapy failure by masking the tumor epitope and blocking the tumor stroma components of breast cancer (38). Meanwhile, Saul et al. found that the delivery of HER2-targeted CAR-T cells to the brain led to a strong antitumor function in breast cancer with brain metastases by the orthotopic xenograft model, which solved the difficulty of drugs breaking through the blood-brain barrier in tumor brain metastasis (39).

### HER3/4

As a heterodimer and signal transduction partner of HER2, HER3/HER4 is related to oncogenic signaling and treatment resistance in breast cancer (61). Heregulin (HRG), a secreted soluble growth factor in cells, contains an epidermal growth factor subdomain and has a high affinity for HER3/4 receptors. It can induce heterodimerization of the HER tyrosine kinase receptor family by binding to specific receptors (62). This extracellular domain of HRG was designed to construct HER3/4-targeted CAR-T cells. Those cells have been found to specifically recognize and have a strong tumor-killing effect on HER3-overexpressing breast cancer cells by *in vitro* experiments and transplanted tumor models (40).

### EGFR

EGFR, one of the important members of the EGFR tyrosine kinases family, is found to be overexpressed in approximately half of TNBC and has a significant regulatory ability in breast cancer progression and malignant transformation (63). The activation of EGFR causes the autophosphorylation of its tyrosine kinase domain by binding to the EGFR receptor and activates downstream PI3K/AKT signaling pathways (64, 65). EGFR-specific CAR-T cell have shown anticancer potential in lung cancer (66), and better safety and anti-tumor effect in phase I clinical trials of pancreatic cancer (67). Xia et al. found that EGFR-targeted CAR-T cells showed a specific and strong tumor-killing ability on TNBC *in vitro*, and this ability was further confirmed in xenograft mouse models. Mechanism studies have confirmed that EGFR-targeted CAR-T cells can activate the granzyme-perforin-PARP and Fas-FADD-caspase signaling pathways in TNBC cells, which may be an important mechanism for increasing the antitumor effect (31). In another study, Liu et al. designed two different types of EGFR-targeted CAR-T cells, which have different DNA sequences in the scFv region. These CAR-T cells can identify TNBC cells with high EGFR expression and trigger TNBC cell death *in vitro* assays and xenograft mouse models (41).

### MSLN

Mesothelin (MSLN) is a glycoprotein on the surface of mesenchymal cells. Its expression has been found to be upregulated in various types of cancers, including breast cancer, making MSLN-targeted CAR-T cells a potential opinion in breast cancer therapy (68, 69). Zhang et al. designed a third-generation MSLN-targeted CAR-T cell containing CD28 and 4-1BB costimulatory domains. In *in vitro* and *in vivo* xenograft models of breast cancer, MSLN-targeted CAR-T cells specifically damaged MSLN-positive breast cancer cell lines and prominently inhibited the growth of breast cancer tumors. Concurrently, T cell and cytokine secretion levels were found to be significantly increased in the presence of CAR-T cells (42, 70).

### ICAM1

Intercellular adhesion molecule-1 (ICAM1) is a type of cell surface transmembrane glycoprotein receptor and a member of the immunoglobulin superfamily. The function of ICAM1 was found to be correlated with tumor cell adhesion, cell growth signaling pathway, and the transport of immune cells to inflammation sites. The expression level of ICAM1 is higher in TNBC than in normal breast tissues (71). Mg2, an ICAM1-specific scFv, was selected as an extracellular antigen-binding domain. *In vitro* tumor cell and TNBC mouse model experiments have revealed that ICAM1-targeted CAR-T cells possess a strong ability to specifically destroy TNBC cells, significantly reduce the growth of TNBC tumors, and improve the survival rate of the mouse model (43).

### AXL

AXL is a type of tyrosine kinase receptor (RTK) originally discovered in patients with chronic myeloid leukemia. AXL is overexpressed in the breast cancer cell membrane, and its overexpression is related to lower survival in patients (72). Previous studies have suggested that it could be implicated in tumor physiological processes, including proliferation, apoptosis, migration, inflammation, and angiogenesis. Moreover, it can activate various intracellular downstream signaling pathways such as NF-κB, MAPK, mTOR, AKT, and PI3K (73, 74). Wei et al. constructed AXL-targeted CAR-T cells and detected their antigen-specific cytotoxicity and cytokine release ability in AXL-positive tumors *in vitro*. The experimental result showed that AXL-targeted CAR-T cells have a significant antitumor ability and stronger persistence in TNBC xenograft models (44).

### MUC1

MUC1 is a type of transmembrane mucin protein that is heavily glycosylated and often expressed on most glandular epithelial cells and organs (75). Overexpression and aberrant glycosylation of MUC1 were found in over 90% of breast cancer patients (76). Abnormally glycosylated MUC1 (tMUC1) can be specifically recognized by synthetic monoclonal antibody TAB004 in breast cancer, but not in normal structured MUC1 (77). Zhou et al. designed the MUC28z chimeric antigen receptor using TAB004 construction as the antigen-binding domain. These types of CAR-T cells enhanced the expression and secretion of cytokines and chemokines such as Granzyme B and IFN-γ after recognizing the tMUC1. tMUC1-targeted CAR-T cells showed significant cytotoxicity and anti-tumor effect and decreased TNBC tumor proliferation and growth *in vitro* and in xenograft models (45, 78).

### GD2

Ganglioside (GD2) is an acidic glycosphingolipid with two sialic acid residues, identified as a marker in breast the stem cell-like cells of breast cancer (79). The expression level of GD2 is upregulated in TNBC (80). Seitz et al. designed a novel GD2-targeted CAR-T cell to recognize and damage GD2-positive tumor cells. The construction of the scFv in the CAR was based on dinutuximab beta, a type of monoclonal antibody CH14.18. This research found that the activation of GD2-targeted CAR-T cells mediated tumor cell death and prevented progression and metastasis in breast cancer (46, 81).

### FRα

Folate receptor α (FRα) is a membrane-binding protein with a high affinity for folic acid, which has the function of transporting folic acid into cells. FRα is overexpressed on the surface of breast cancer cells, but not in normal tissues, making it a promising targeting antigen in breast cancer (82). Luangwattananun et al. generated FRα-targeted CAR-T cells by the lentiviral system. These specific CAR-T cells have a significant antitumor ability when co-cultured with TNBC cells expressing FRα. Moreover, its cytotoxic effect was more obvious in cell with increased FRα expression and not observed in FRα-negative normal breast cells. Concurrently, CAR-T cells did not produce this specific cytotoxicity on FRα-negative MCF10A normal breast cells (32).

### PD-L1

Programmed death receptor 1 (PD-1, CD279) and programmed death ligand 1 (PD-L1) can activate immune cell inhibitory signals, and their expression is usually upregulated in tumor patients with continuous T cell activation (83). Targeting PD-L1 is a promising target and has achieved good results in clinical trials in a variety of tumors (84, 85). A chimeric PD-1 (chPD1) receptor has been developed, which can recognize PD-L1 expressed in breast cancer. Parriott et al. designed ChPD1-T cells for recognizing and damaging tumor cells by secreting inflammatory factors such as IL2, IL-17, IL-21, IFN γ, TNF, and GM-CSF and decreasing the inflammatory suppressor cytokine IL-10. ChPD1-T cells significantly reduced the tumor burden and prolonged tumor-free survival in tumor-bearing mice (47). Bajor et al. found that the PD-L1-targeted CAR-T cells showed a strong degranulation response and cytokine production in TNBC cells with a higher expression of PD-L1. The co-culture of low PD-L1-expressing tumor cells and CAR-T cells can result in delayed tumor cell clearance by inducing PD-L1 expression on tumor cells. Further research confirmed that HER-2-targeted CAR-T cells could enhance the expression level of PD-L1 on breast cancer cells, synergistically increasing the tumor-killing function of PD-L1-targeted CAR-T cells (48).

### PTK7

Protein Tyrosine kinase 7 (PTK7), an important member of the receptor tyrosine kinases (RTKs) family, has an intracellular domain structure that catalyzes inactive tyrosine kinase (86). The expression of PTK7 has been shown to be increased in breast cancer (87). Three different types of PTK7-specific CARs (PTK7-CAR1/2/3) were constructed, all of them containing an artificial modified PTK7-specific scFv domain, CD8α molecules transmembrane domain, CD3ζ intracellular domain sequences, and 4-1BB intracellular costimulatory domain. These CAR-T cells all led to increased cytokine production and cytotoxicity to high PTK7-expressing breast cancer without causing obvious damage to normal tissue (49).

### Trop2

Trophoblast cell surface antigen 2 (Trop2), a cell surface glycoprotein, is overexpressed in TNBC and has a significant function in tumor growth, proliferation, migration, and metastasis (88). Chen et al. developed a novel Trop2-targeted CAR-T cell. These constructed CAR-T cells showed a strong tumor-killing ability in breast cancer cells expressing Trop2 by *in vitro* experiments. The addition of CD27 in Trop2-targeted CAR-T cells increased their antitumor effect in tumor cells and tumor-bearing mouse models by enhancing the expression of IL-7Rα and reducing the expression of PD-1 (25).

### SLC3A2

Ansari et al. found that higher expression of the tumor-associated antigen SLC3A2, a cell surface protein, played a significant role in tumor metabolism and predicted a worse prognosis in breast cancer (89). SLC3A2-targeted CAR-T cells have shown cytotoxicity against breast cancer tumor cells by simultaneously stimulating the production of INF-γ and IL-2 production *in vitro*. In an *in vivo* xenograft model, SLC3A2-targeted CAR-T cells significantly improved overall survival and reduced subcutaneous xenograft tumor growth and tumor burden without weight loss and cytokine release syndrome (CRS) (50).

### B7-H3

B7-H3 is an immune checkpoint molecule also regarded as CD276, which is part of the B7 superfamily of immune checkpoint inhibitors (90, 91). Chen et al. revealed that B7-H3 was overexpressed in breast cancer, and that upregulation of B7-H3 was correlated with poor prognosis and clinical outcomes in breast cancer, implying that B7-H3 could be a prospective target for CAR-T therapy (92). Lei et al. found that B7-H3-targeted CAR-T cells could specifically damage B7-H3-expressing solid tumor cells, including breast cancer. Meanwhile, a low dose of SAHA, an inhibitor of histone deacetylases, significantly increased the antitumor effect of B7-H3-targeted CAR-T cells *in vitro* by enhancing the expression of B7-H3 and reducing the secretion of CTLA-4 and TET2 with their immunosuppressive function (51).

### CD70

CD70, a key member of the necrosis factor receptor superfamily, is expressed on the cell surface and widely overexpressed in a variety of tumors (93, 94). Yang et al. designed a type of bivalent tandem CAR (TanCAR) both targeting CD70 and B7-H3 molecules. The modified CAR-T cells can specifically bind to CD70 and have a higher persistence and antitumor capacity on CD70-positive breast cancer cells. TanCAR-T cells increased the capacity to induce tumor cell damage and cytokine release in breast cancer cells compared to single-chain specific CAR-T cells when they were applied to breast cancer cells expressing both target antigens (35).

### VEGFR 2/3

Vascular endothelial growth factor (VEGF) and vascular endothelial growth factor receptor (VEGFR) have a crucial physiological function in angiogenesis and lymphangiogenesis, which are closely associated with tumor cell molecular and biological functions including growth, invasion, migration, and metastasis (95, 96). Blocking or interfering with the interaction between VEGF and VEGFR has become a possible method for tumor therapy. VEGFR-2 and VEGFR-3 are important members of the VEGFR family, and VEGFR-2 or VEGFR-3-targeted CAR-T cells were designed to verify their potential in the treatment of breast cancer. Xing et al. found that these CAR-T cells exhibited strong cytotoxicity against both VEGFR-2/3-positive breast cancer cells by up-regulating the production capacity of INF-γ, TNFα, and IL-2 cytokines. Moreover, VEGFR-2/3-targeted CAR-T cells significantly inhibited the proliferation, invasion, and metastasis capacity of xenograft tumors in nude mice models and disrupted the tubular structures of endothelial cells (52).

### TEM8

Tumor endothelial marker 8 (TEM8), a glycoprotein with highly conserved integrin, is involved in endothelial cell invasion and metastasis and is initially regarded as a tumor endothelial marker (97). The expression of TEM8 is elevated in breast cancer cells, and higher expression of TEM8 is associated with higher growth, metastasis, and recurrence rates of breast cancer (98). TEM8-targeted CAR-T cells can secrete immune-stimulating cytokines and block tumor angiogenesis by damaging TEM8-overexpressing TNBC cells and tumor vascular endothelial cells after TEM8-specific recognition. These cells can also induce the regression of TNBC-derived xenograft tumors and counteract the formation of mammary globules by targeting stem cell-like breast cancer cells (53).

### NKG2DLs

NKG2D (Natural Killer Group 2, member D) is a type of receptor highly expressed in NK cells and T cells. NKG2D ligands (NKG2DLs) are frequently upregulated in multiple tumor cells, including breast cancer cells. The combination of NKG2D in immune cells and NKG2DLs on tumor cells plays a significant role in the activation of their tumor-killing effect in immune cells (99). *In vitro*, NKG2DLs-targeted CAR-T cells could effectively recognize and eliminate TNBC overexpressing NKG2DLs. Furthermore, the costimulatory domains with 4-1BB or CD27 molecule specifically enhanced the persistence of CAR-T cells (30, 100).

### αvβ6 integrin

The integrin αvβ6, a member of the heterodimeric cell surface receptors family, mediates cell-cell and cell-extracellular matrix interactions. The αvβ6 integrin was up-regulated in breast cancer and its overexpression correlated with the prognosis of cancer patients (101). The integrin αvβ6 could activate the TGFβ signaling pathway and promote cell proliferation and migration, epithelial-mesenchymal transition, and matrix metalloproteinase activity (102, 103). A highly selective αvβ6-targeted CAR-T cell was constructed by combining the fused CD28^+^CD3 domain with the A20 peptide derived from the foot-and-mouth disease virus. IL-4-responsive fusion gene (4αβ) was co-expressed in CAR-T cells to increase the proliferation and expansion ability and persistence of these cells *in vivo*. Whilding et al. found that αvβ6-targeted CAR-T cells exhibited strong cytotoxicity to breast cancer cells with less damage to normal tissues *in vivo* and *in vitro* (54, 104).

### CD32A^131R^

Antibody-dependent cell-mediated cytotoxicity (ADCC) is a common method by which the immune cells kill tumor cells. The recognition and dissolution process of tumor cells is affected by the affinity with which the Fc fragment of the antibody binds to the FcγR domain of immune effector cells. CD32, a member of the FcγR family, is composed of three different variants A, B, and C which have affinities for Fc segments (105). CD32A^131R^ was defined based on arginine at position 131. Caratelli et al. designed a low-affinity chimeric receptor CD32A^131R^ to induce the elimination of EGFR-overexpressing breast cancer by crosslinking with cetuximab. These CAR-T cells could effectively recognize specific cetuximab-bound tumor cells and promote the expression and secretion of INF γ and TNFα by combining cetuximab and CAR-T cells (55).

## Combination therapy with CAR-T

Although there have been significant advances in CAR-T cell therapy in solid tumors, the efficacy of CAR-T cells alone in solid tumors treatment remains limited. Consequently, effective approaches to promote CAR-T cells therapy are still needed. Several studies have shown that the persistence and tumor-killing ability of CAR-T cells are influenced by numerous molecules or genes expression. We summarize several prospective approaches for combining CAR-T cells with other molecules to improve therapeutic efficacy.

### Combination therapy in HER2-targeted CAR-T

Il-21 is a cytokine in the TME that can promote T cell proliferation and drive the T cell memory effect and has the function of preventing tumor metastasis or recurrence (106). Du et al. found that IL-21 can augment the aggregation and amplification capacity of poorly differentiated CAR-T cells and effectively increase the cytotoxicity of HER2-targeted CAR-T cells to HER2-overexpressing cells by increasing cytokine secretion in breast cancer. Their study demonstrated that the addition of IL-21 significantly increased strong cytotoxicity against trastuzumab-resistant breast cancer cells with the synthesis and secretion of IFN-γ and IL-2, after combining HER2-targeted CAR-T cells with trastuzumab-resistant HCC1954 and BT474 cells (107).

Furthermore, Li et al. found that the anti-PD1 antibody can enhance the therapeutic effect of HER2-targeted CAR-T cells (108). In another study of homologous mouse models, more HER2-targeted CAR-T cells were shown to reside in the tumor stroma with the addition of an anti-PD1 antibody, significantly increasing the ability to recognize tumors and maintain T cell persistence. The results suggested that the anti-PD1 antibody can increase the tumor-killing ability of CAR-T cells and reduce the tumor weight (109).

The IKZF family proteins contain a zinc finger domain that can recognize specific DNA sequences, bind other proteins, and activate or inhibit targeted genes by reshaping chromatin and binding to RNA Pol II transcription initiation complexes (110). The knockout of transcription factor IKZF3 in HER2-targeted CAR-T cells can significantly improve the ability to kill cancer cells by increasing T cell activation and proliferation without affecting the activity and function of CAR-T cells (111).

### Combination therapy in EGFR-targeted CAR-T cells

Polyinosinic-polycytidylic acid (poly I: C) is a type of synthetic double-stranded RNA (dsRNA) analog. It can be recognized and bound by toll-like receptor 3 (TLR3) and protein kinase (PKR) activated by dsRNA. It may mediate immune functions and has extensive antitumor effects on a variety of cancers (112, 113). The joint application of EGFRVIII-targeted CAR-T cells with Poly I: C prominently increased the tumor-killing ability of CAR-T cells against tumor cells and promoted the production and secretion of IFN γ and IL-2. It also improved the tumor growth and proliferation inhibitory effect of CAR-T cells in subcutaneous breast cancer-transplanted mice. Meanwhile, this composition resulted in a huge decrease in the number of myeloid-derived suppressor cells (MDSC) in the spleen and peripheral blood, which may reduce the immunosuppressive effect of MDSC in the tumor immune process (114).

Olaparib, an oral poly ADP-ribose polymerase (PARP) inhibitor, was shown to have clinical benefits against mutated BRCA-positive metastatic breast cancer (115). Sun et al. found that olaparib could prominently increase the antitumor effect of EGFRVIII-targeted CAR-T cells by inhibiting the migration and aggregation of MDSC and promoting the survival and persistence of T cells in the TME. Mechanistically, olaparib was shown to decrease the migration of MDSC by preventing the expression of SDF1α released by cancer-associated fibroblasts (CAFs), increasing the immune effect of CAR-T cells on the tumor (116).

CDK7 is a key component of the transcription factor TFIIH, which is introduced to the transcription initiation site adjacent to PolII to promote the initiation of transcription (117). Xia et al. found that EGFR-targeted CAR-T cells-resistant breast cancer cells are particularly susceptible to THZ1, a CDK7 inhibitor. The combination of THZ1 and EGFR-targeted CAR-T cells exhibit a better ability to inhibit immune resistance and prevent tumor proliferation and metastasis processes compared to applying to CAR-T cells alone in TNBC tumor models (118).

### Combination therapy in ROR1-targeted CAR-T cells

Receptor tyrosine kinase-like orphan receptor 1 (ROR1), a type I transmembrane receptor in the ROR family, has an extracellular ligand-binding domain and an intracellular tyrosine kinase domain (119). Nicholas et al. summarized that ROR1 was involved in inhibiting cell apoptosis, enhancing the EGFR signaling pathway, and inducing tumor epithelial-mesenchymal transformation (EMT) (120). However, ROR1-targeted CAR-T cells showed limited efficacy in breast cancer. Srivastava et al. found that oxaliplatin can activate tumor macrophages and release T cell recruitment chemokines, which could improve ROR1-targeted CAR-T cell infiltration. Moreover, oxaliplatin combined with anti-PD-L1 can synergistically improve the function of damaging tumors by ROR1-targeted CAR-T cells (56).

Transforming growth factor (TGF) β is one of the commonly accepted immunosuppressive cytokines in the TME and is correlated with the antitumor effect of ROR1-targeted CAR-T cells. Cytokine production and the proliferation function of ROR1-targeted CAR-T cells were prominently impaired in the presence of TGF-β. Tanja et al. found that blocking the TGF-β receptor signaling by inhibitor SD-208 can promote the tumor-killing function of ROR1-targeted CAR-T cells (121).

### Combination therapy in other CAR-T cells

Interleukin-7 receptor (IL7R) is present on the surface of lymphoid progenitor cells surface and is essential for normal lymphocyte development (122). The up-regulation of IL7 or the IL-7 receptor was found to prolong the persistence of immune cells and enhance antitumor effects (123). Zhao et al. found that the activation of the IL-7 receptor could enhance the antitumor function and prolong the survival time of traditional AXL-targeted CAR-T cells by increasing the growth, proliferation, activation, and cytotoxicity capacity of CAR-T cells *in vitro* and *in vivo* (124).

The P38 pathway, a stress-activated protein kinase pathway, is also often disrupted and associated with cancer survival and migration in humans (125). By CRISPR-Cas9 screens and functional testing of T cells, it was found that interfering in the P38 pathway could enhance the expansion ability and limit the oxidation and differentiation pressures of T cells. P38 inhibitors (P38i) were found to cause CAR-T cells to be more effective in T cell-mediated tumor-specific lysis, manifesting the promising clinical application of P38i in increasing the antitumor function of CAR-T cells (126).

The persistence ability of CAR-T cells in the TME is an important factor in impeding their therapeutic effect in solid tumors (127). The CD8^+^ T 17 (Tc17) cells and T helper 17 (Th17) cells were found to be more persistent in the TME (128). A study has found that CAR-T cells produced by Th/Tc17 cells could improve the persistence ability and tumor-suppressive role of CAR-T cells in the TME when the stimulator of STING agonists DMXAA or cGAMP were combined with anti-PD-1 antibodies. Single-cell RNA sequencing demonstrated that DMXAA could promote the transport of CAR-T cells and regulate their immune effect in the TME by producing a chemokine (129).

The rAd.sT is a type of oncolytic adenovirus targeting TGF-β signaling. Li et al. reported that the combination of MSLN-targeted CAR-T cells and rAd.sT in breast cancer therapy can increase the production of cytokines IL-6 and IL-12 in the TME, resulting in a stronger tumor inhibition effect (130).

## Clinical Trials in breast cancer

In several years of research on breast cancer, human trials of some prospective targets of CAR-T cells have been confirmed all over the world to verify the clinical treatment effectiveness and safety of the therapy. Specific clinical trials are summarized in **Table 2**. In 2017, a Phase I clinical trial (NCT01837602) with c-Met-targeted CAR-T cell was conducted in metastatic breast cancer patients. The mRNA of CAR was detected in the blood of 2 patients and tumor tissue of 4 patients, and cell injection was well tolerated, with no grade greater than 1 associated adverse reaction. The tumor was killed and immunohistochemical analysis showed that the CAR-T cells caused an inflammatory response within the tumor, resulting in extensive tumor necrosis (131). However, another phase 1 clinical trial targeting c-Met CAR-T (NCT03060356) was terminated due to the halt in funding. By far, the CAR-T cell therapy clinical trials targeting HER 2 were conducted the most widely in breast cancer patients. There has been a total of five phase 1/2 clinical trials. Two of them have been withdrawn (NCT02547961) (NCT02713984) and three are being recruited (NCT04650451) (NCT03740256) (NCT04430595). In addition, the phase 1 clinical studies on MUC1-targeted CAR-T cells are also widely carried out. (NCT02587689) (NCT04020575) (NCT04025216). Phase 1/2 clinical trials of CAR-T cells targeting CEA in the CET-positive breast cancer are also ongoing (NCT02349724) (NCT04348643). Moreover, there are several other clinical trials targets, including EpCAM (NCT02915445), NKG2DL (NCT04107142), ROR1 (NCT02706392), CD70 (NCT02830724), C7R/GD2 (NCT03635632), CD133 (NCT02541370) and CD44v6 (NCT04427449). In the future, more CAR-T cell clinical treatment trials for breast cancer patients will still be carried out to obtain the best therapeutic effect.

**Table 2:** The summary of clinical trials of CAR-T cell therapy in breast cancer.

Targeting antigen	Study Title	Estimated Enrollment	Phase	Indication	Clinical Trials ID	Status
HER-2	Chimeric Antigen Receptor-Modified T Cells for Breast Cancer	0	Phase 1/2	Breast Cancer	NCT02547961	Withdrawn
EpCAM	EpCAM CAR-T for Treatment of Nasopharyngeal Carcinoma and Breast Cancer	30	Phase 1	Recurrent Breast Cancer	NCT02915445	Recruiting
MUC1	Autologous huMNC2-CAR44 T Cells for Breast Cancer Targeting Cleaved Form of MUC1 (MUC1)	69	Phase 1	Metastatic Breast Cancer	NCT04020575	Active, not recruiting
HER2/GD2/CD44v6	Multi-4SCAR-T Therapy Targeting Breast Cancer	100	Phase 1/2	Breast Cancer	NCT04430595	Recruiting
cMet	cMet CAR RNA T Cells Targeting Breast Cancer	6	Phase 1	Metastatic Breast Cancer/Triple Negative Breast Cancer	NCT01837602	Completed
MLSN	T-Cell Therapy for Advanced Breast Cancer	186	Phase 1	Breast Cancer	NCT02792114	Active, not recruiting
NKG2DL	Haplo/Allogeneic NKG2DL-targeting Chimeric Antigen Receptor-grafted γδ T Cells for Relapsed or Refractory Solid Tumor	10	Phase 1	Triple Negative Breast Cancer	NCT04107142	Unknown
MUC1	Phase I/II Study of Anti-Mucin1 (MUC1) CAR T Cells for Patients with MUC1+ Advanced Refractory Solid Tumor	20	Phase 1/2	Triple Negative Breast Cancer	NCT02587689	Unknown
cMET	Autologous T Cells Expressing MET scFv CAR (RNA CART-cMET)	77	Phase 1	Breast Cancer	NCT03060356	Terminated
HER2	Safety and Activity Study of HER2-Targeted Dual Switch CAR-T Cells (BPX-603) in Subjects with HER2-Positive Solid Tumors	220	Phase 1	HER2-positive Breast Cancer	NCT04650451	Recruiting
CEA	A Clinical Research of CAR T Cells Targeting CEA Positive Cancer	75	Phase 1	Breast Cancer	NCT02349724	Unknown
CEA	Safety and Efficacy of CEA-Targeted CAR-T Therapy for Relapsed/Refractory CEA+ Cancer	40	Phase 1/2	Breast Cancer	NCT04348643	Recruiting
TnMUC1	A Study of CART-TnMUC1 in Patients with TnMUC1-Positive Advanced Cancers	112	Phase 1	Triple Negative Breast Cancer	NCT04025216	Recruiting
HER-2	A Clinical Research of CAR T Cells Targeting HER2 Positive Cancer	0	Phase 1/2	Breast Cancer	NCT02713984	Withdrawn
ROR1	Genetically Modified T-Cell Therapy in Treating Patients with Advanced ROR1+ Malignancies	60	Phase 1	Stage IV Breast Cancer	NCT02706392	Recruiting
CD70	Administering Peripheral Blood Lymphocytes Transduced with a CD70-Binding Chimeric Antigen Receptor to People with CD70 Expressing Cancers	2	Phase 1/2	Breast Cancer	NCT02830724	Suspended
HER2	Binary Oncolytic Adenovirus in Combination with HER2-Specific Autologous CAR VST, Advanced HER2 Positive Solid Tumors (VISTA)	45	Phase 1	Breast Cancer	NCT03740256	Recruiting
MSLN	Treatment of Relapsed and/or Chemotherapy Refractory Advanced Malignancies by MSLN targeted CAR-T	20	Phase 1	Triple Negative Breast Cancer	NCT02580747	Unknown
C7R/GD2	C7R-GD2.CART Cells for Patients with Relapsed or Refractory Neuroblastoma and Other GD2 Positive Cancers (GAIL-N)	94	Phase 1	Breast Cancer	NCT03635632	Recruiting
CD133	Treatment of Relapsed and/or Chemotherapy Refractory Advanced Malignancies by CART133	20	Phase 1/2	Breast Cancer	NCT02541370	Completed
CD44v6	4SCAR-CD44v6 T Cell Therapy Targeting Cancer	100	Phase 1/2	CD44v6-Positive Breast Cancer	NCT04427449	Recruiting

## Challenges of CAR-T cell therapy

**Figure 4:**
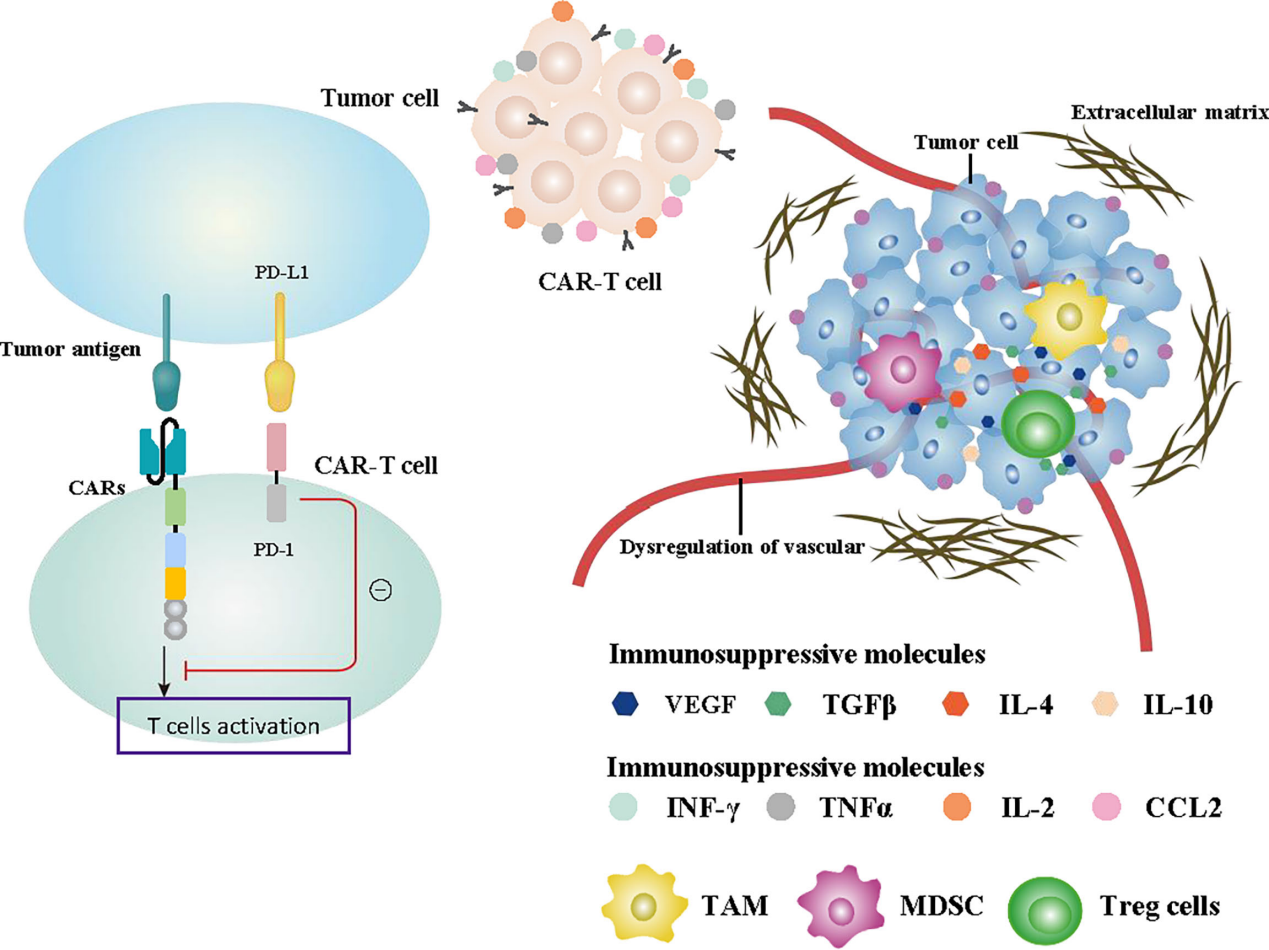
Summary of the challenges affecting CAR-T cell function. Specifically designed CAR structures and the production and release of some cytokines promoting immune function such as INF-γ, TNFα, IL-2, and CCL2 effectively enhanced the antitumor ability of CAR-T cells. The recruitment of immunosuppressive cells such as TAMs, MDSCs, and Treg cells, the release of immunosuppressive cytokines including TGFβ, VEGF, IL-4, and IL-10 in the TME, and the activation of PD-1 signaling all inhibit tumor immunity. Abnormal formation of extracellular matrix and dysregulation of the vascular system are also important factors affecting CAR-T cells.

Although CAR-T therapy for solid tumors has been widely studied in the laboratory and clinic and has shown good progress, its clinical efficacy remains unsatisfactory. Severe adverse reactions, tumor cell heterogeneity, immunosuppression in the TME, and the persistence of CAR-T cells are the obstacles faced in CAR-T cell therapy, which are shown in **Figure 4**. Therefore, better engineering strategies should be developed in future research to improve the clinical efficacy of and minimize adverse reactions in CAR-T cell therapy. In the following, we summarize the problems and possible solutions of CAR-T cell therapy.

### Specific target

Due to the specific mechanism and characteristics by which CAR-T cells recognize and damage tumor cells, it is very important to identify specific tumor targets expressed on the tumor cells’ surface and that have higher expression levels in tumor tissue than in normal tissue. In the previous section, we summarized some potential specific target molecules in breast cancer. However, most targets are only effective against specific types of breast cancer on account of the heterogeneity of tumor cells. Concurrently, the escape of target antigens, characterized as the complete or partial loss of tumor antigens, is another huge problem. Although CAR-T cells possessed a high initial response rate to tumor cells, a significant reduction in response rate was reported in a substantial proportion of patients who injected CAR-T cells repeatedly (132).

The joint identification of multiple targets is an alternative mean of overcoming antigen escape. Bivalent tandem CAR (TanCAR) targeting both CD70 and B7-H3 has shown the ability to enhance tumor recognition by CAR-T cells (35). Meanwhile, a phase 1 clinical trial (NCT04430595) combined with HER2, GD2, and CD44v6 targets are currently underway, which can effectively reduce antigen escape and increased recognition of tumor cells.

### Treatment-related toxicities

Neurotoxicity and cytokine release syndrome (CRS) are the two most common severe and unpredictable reactions to CAR-T cell therapy. Current studies suggest that these adverse reactions are associated with the high level of cytokines secreted by CAR-T cells (133). Neurotoxicity usually presents with seizures, delirium, memory loss, and acute cerebral edema, while CRS typically presents with fever, hypotension, and respiratory insufficiency (134, 135). In a case of metastatic colon cancer, the patient had respiratory distress within 15 min after HER2-targeted CAR-T cell injection and died 5 days later. The chest radiograph and serum samples showed significant immune infiltration in the lung. It is speculated that numerous CAR-T cells migrate to the lung immediately after infusion and trigger cytokine release by recognizing the low levels of HER2 on the lung epithelial cells. The expression of immune cytokines including IL-6, TNF-α, IL-10, GM-CSF, and IFN-γ was significantly increased. The cytokine storm caused by CAR-T treatment could result in respiratory distress and death (136). Although this was a case report about severe adverse reactions caused by HER2-targeted CAR-T cell therapy for colon cancer, effectively avoiding damage to normal lung tissue is still a problem that needs to be discussed and solved, as HER2-targeted CAR-T is widely used in breast cancer treatment. Therefore, we believe that a serious cytokine storm can also occur in breast cancer treatment.

In the research of CAR-T exosomes, purified exosomes from CAR-T cells were found to express perforin, granzyme B, and cell membrane molecules including CARs, CD3, CD8, and TCRs. The application of CAR-T exosomes in the treatment of breast cancer can effectively control toxicity and improve safety (137). Furthermore, Sterner et al. and Giavridis et al. found that IL-1, IL-6, and GM-CSF participated in the CRS regulation process, and the knockdown of cytokine coding genes or specific cytokine inhibitors may significantly decrease the occurrence rate of CRS (138–140). Moreover, suicide genes, which could lead to cell death through a small molecule-mediated activation process, have been introduced in CAR-T cells as a new possible mechanism to avoid the unpredictable therapeutic reactions of CAR-T cells. The fusion of the modified caspase 9 protein into the human FK506 binding protein (FKBP) can effectively and specifically eliminate CAR-T cells expressing the suicide gene without affecting the growth and proliferation of normal CAR-T cells, which reduces damage to normal tissue (141). Glucocorticoid is a potent anti-inflammatory drug that effectively relieves patients’ brain inflammation and vasogenic edema symptoms due to CAR-T cell therapy (142). Kloss et al. found that the recognition of different antigens on tumor cells by CAR-T cells can effectively increase the specificity of tumor recognition and reduce the damage to normal cells (143). Based on this opinion, Srivastava et al. designed logic-gated ROR1-targeted CAR-T cells. The synthetic Notch (synNotch) receptors were designed to recognize EpCAM or B7-H3 on the tumor. ROR1 CAR expression is induced by synNotch receptor activation. In breast cancer studies, this strategy mediates antitumor effects on ROR1^+^ breast cancer without toxicity reaction to normal tissues (144).

### The proliferation and persistence of CAR-T cells

The proliferation and persistence ability of CAR-T cells are often directly related to the antitumor effect. The function of some genes in CAR-T cells affects their persistence. As mentioned above, the addition of costimulatory domains to the structure of CARs was the traditional method of significantly increasing the proliferation and persistence of CAR-T cells (30). Agnes et al. found that colony stimulating factor-1 (CSF-1) was associated with immune cell proliferation by binding to the CSF-1 receptor, which was encoded by a c-fms gene in the cancer cells. The c-fms gene was expressed in T cells by gene-modified. The addition of CSF-1 stimulated the proliferative effect of CAR signals by the secretion of IFN and IL-2 without compromising the cytotoxicity in these gene modification cells (145). In another study, Boucher et al. reported that CAR-T cells with mutated CD28 subdomains had better survival and function. The expression of various genes relevant to T cell depletion, such as Nfatc1, Nr42a, and Pdcd1, were significantly reduced through null mutations of the CD28 subdomain (146). In addition, several transcription factors are known as inductors of T cell exhaustion. Khan et al. found that TOX can transform effector T cells with antitumor function into non-functional exhausted T cells by driving epigenetic remodeling of exhausted T cells (147). TCF-1 is another transcription factor that could regulate the transformation of exhausted T cells by mediating the expression of Eomes and c-Myb (148). The activation of transcription factor NR4A is related to the expression of immunosuppressive molecules such as PD-1 and TIM3. CAR-T cells showed stronger tumor-killing activity and better persistence with the knockout of NR4A (149). Furthermore, the poorly differentiated T cell subsets including stem cell memory T (TSCM) cells, naive T cells, and central memory T (TCM) cells have a high proliferative capacity. The use of these to design CAR-T cells is an effective method to prolong the proliferation and persistence and enhance the antitumor activity of CAR-T cells in patients when constructing CAR-T cells (150).

### Immunosuppressive effect of the tumor microenvironment

Immune evasion is a major challenge in antitumor immunotherapy, which directly determines the effectiveness of tumor immunity. The change of cytokines and chemokines in the TME is an important factor affecting immune escape. The process of antitumor immune activation releases numerous cytokines. The expression of the chemokine pattern in the TME has changed to preferentially recruit and inhibit inflammation cell types and avoid recruiting antitumor immune cells. This physiological process resulted in numerous immunosuppressive cells existing in the TME, which suppressed the antitumor function of immune cells (151–153). Immunosuppressive cells, including MDSCs, TAMs, and Treg cells, are recruited by cytokines in the TME, which is a key reason for the immunosuppression effect (154–158). In addition, Binnewies et al. suggested that chemokines and cytokines, including IL-4, IL-10, TGFβ, and VEGF in the TME can directly suppress the T cell effect and improve the aggregation of immune inhibitory cells. Concurrently, the assembled inhibitory cells also secrete numerous immunosuppressive cytokines, which further enhance the immunosuppressive effect with a positive feedback process (159).

It is a common method to enhance the immune response of CAR-T cells by promoting the expression of immune-enhancing genes and cytokines secretion. Adachi et al. found that upregulation of IL-7 and CCL19 genes in CAR-T cells may improve the invasion of T cells or dendritic cells in solid tumor tissue in mouse models, promoting tumor regression (160). Moreover, the overexpression of IL-18 and IL-12 genes in CAR-T cells could activate endogenous immune cells and enhance antitumor responses (161). Research has shown that cytokines such as INF-γ, TNFα, and IL-2 can enhance the anti-tumor function of CAR-T cells (52).

The activation of T cell immunosuppressive signal in the TME is an important obstruction in CAR-T cells therapy. PD-1/PD-L1 is one of the most characterized and studied signals in breast cancer. Previous research has shown that the inhibition of PD-1 signaling has been shown to produce significant clinical benefits in various tumor patients, including breast cancer. The activation of the PD-1 signal induces the depletion and inactivation of CAR-T cells (9). The anti-PD1 antibodies significantly improved the antitumor role of targeting HER2 CAR-T cells (108).

### Physical barriers

Cancer-associated fibroblasts (CAF) are stromal cells in the TME. They could promote the deposition of abnormal extracellular matrix (ECM) around the tumor to form a dense fibrotic environment and limit CAR-T cell transport to tumor tissues. The tumor immunosuppression effect induced by CAF is an obstacle to promoting the therapeutic efficacy of CAR-T cells (162). The activation process of TGF-β could enhance the secretion of ECM proteins by CAF, leading to the formation of a physical network and restricting the movement of T cells. Although previous studies have confirmed that HER2-targeted CAR-T cells have a stronger ability to penetrate ECM than traditional antibody drugs (38), the presence of ECM in the TME can still hinder the ability of CAR-T cells to recognize and kill tumors.

NOX4 is a downstream molecule of TGFβ signaling. The inhibition of NOX4 not only blocks TGF-β signaling but also prevents CAF differentiation, which reduces EMC protein secretion and promotes immune cell infiltration into tumor tissues (163, 164). Caruana et al. reported that the lack expression of the enzyme heparinase (HPSE) after *in vitro* manipulation of T cells may be responsible for the reduced ability to degrade and penetrate the ECM. HPSE can degrade heparin sulfate proteoglycan, the main component of ECM. GD2-targeted CAR-T cells expressing HPSE were designed to enhance the ability to degrade ECM and promote T cell invasion and antitumor activity to tumors, including breast cancer cells (165).

### Dysregulation of the vascular system

The vascular system of tumors often undergoes remodeling and shows severe vascular abnormalities and dysfunction. The penetrability of immune cells from blood vessels to solid tumors is impaired, leading to the diminished antitumor effect of CAR-T cells (166). Vascular system dysregulation and inadequate endothelial energy in tumor tissues downregulate the expression level of intercellular adhesion molecule 1(ICAM1) and adhesion molecules VCAM1, which limits T cell infiltration in tumor tissues (167). Meanwhile, normalization of the tumor vascular system can enhance tissue blood perfusion to improve the infiltration and viability and promote the antitumor ability of CAR-T cells.

Bevacizumab, a humanized monoclonal antibody against VEGF, not only inhibits the germination of new blood vessels but also normalizes the vascular system. Meanwhile, bevacizumab was found to inhibit the down-regulation process of cell adhesion molecules and increase the invasion capacity of CAR-T cells in the tumor (168). Xing et al. designed targeted VEGFR2/3 CAR-T cells and achieved remarkable results in the treatment of breast cancer, which provides a new idea for both antitumor formation and anti-angiogenesis (52). Injecting CAR-T cells into the tumor location directly, keeping them away from the vascular transport system, is another effective treatment strategy. The intraperitoneal injection of CAR-T cells in mesenchymal mesothelioma induced better response and tolerability compared with intravenous injection, which provides a new prospect for local injection therapy of CAR-T cells (169).

## Future outlook

The successful application of CAR-T cells in hematological tumors has made it a promising approach for solid cancer therapy. Moreover, the FDA approval of CAR-T cells for clinical application has greatly facilitated the exploration of CAR-T cell therapy in treating solid tumors. Several CAR-T cells targeting breast cancer-related antigens have been manufactured to exert better therapeutic effects against breast cancer. Although CAR-T cell therapy has demonstrated remarkable antitumor function both *in vitro* and *in vivo*, the severe treatment-related toxicity, less T cell persistence, and immunosuppression in the TME continue to hinder its clinical application in breast cancer and other solid tumors.

Furthermore, it is worth noting that molecule-targeted therapies including monoclonal antibodies and ADC have also achieved excellent results in breast cancer (170). In addition, the preparation and clinical application of these types of drugs were more convenient compared to CAR-T cell therapy. However, the killing effect of monoclonal antibodies such as trastuzumab and pertuzumab on HER2-overexpressing breast cancer cells still depends on the activity of immune cells, and drug resistance due to epitope masking and steric hindrance remains a major difficulty that needs to be addressed appropriately (171). Moreover, ADC depended on antibodies to recognize tumor cells and carry cytotoxic drugs to kill tumor cells. Specific recognition of tumor cells, antibody-drug decoupling, and drug resistance still hinder the widespread use of ADC drugs in breast cancer (172). CAR-T cells can directly recognize and damage tumor cells, which can effectively avoid the difficulties faced in antibody therapy. Hence, CAR T cell therapy remains a promising and indispensable treatment for breast cancer and more engineering strategies are required to enhance the safety and efficacy in future studies.

## Author contributions

HX contributed significantly to fund support and the conception of the review. HZ and WD wrote the manuscript. HZ and SZ were responsible for drawing the figures. RL and H-TZ contributed to proposing some constructive suggestions and revising the manuscript. All authors read and approved the manuscript.

## Funding

This research was supported by the Chinese Society of Clinical Oncology Foundation of Hengrui Medicine (grant number Y-HR2017-055, Y-HR2018-262).

## Conflict of interest

The authors declare that the research was conducted in the absence of any commercial or financial relationships that could be construed as a potential conflict of interest.

## Publisher’s note

All claims expressed in this article are solely those of the authors and do not necessarily represent those of their affiliated organizations, or those of the publisher, the editors and the reviewers. Any product that may be evaluated in this article, or claim that may be made by its manufacturer, is not guaranteed or endorsed by the publisher.
